# [Retracted] Rapamycin suppresses the PI3K/AKT/mTOR signaling pathway by targeting SIRT1 in esophageal cancer

**DOI:** 10.3892/etm.2023.12288

**Published:** 2023-11-06

**Authors:** Tao Liu, Xiangsen Liang, Yu Sun, Shengzhuang Yang

Exp Ther Med 22:1190, 2021; DOI: 10.3892/etm.2021.10624

Following the publication of this paper, it was drawn to the Editor’s attention by a concerned reader that the tumour images shown in Fig. 5B on p. 8 were strikingly similar to data appearing in different form in other articles written by different authors at different research institutes, which had either already been published or were under consideration for publication at around the same time. Owing to the fact that the contentious data in the above article had already been published prior to its submission to *Experimental and Therapeutic Medicine,* the Editor has decided that this paper should be retracted from the Journal. After having been in contact with the authors, they agreed with the decision to retract the paper. The Editor apologizes to the readership for any inconvenience caused.

